# Coffee bean sign, whirl sign and bird's beak sign in the diagnosis of sigmoid volvulus

**DOI:** 10.11604/pamj.2014.19.56.5142

**Published:** 2014-09-23

**Authors:** Mehmet Yigit, Kenan Ahmet Turkdogan

**Affiliations:** 1 Bezmialem Vakif University, Department of Emergency Medicine, Istanbul, Turkey

**Keywords:** Coffee bean, sigmoid volvulus, whirl

## Image in medicine

The patient, a 28-year-old man who had presented to our Emergency Department (ED) with constant abdominal pain and distension for one day, had no previous medical or surgical history. He denied that he had any nausea, vomiting, diarrhea or constipation. At admission, his physical examination revealed hypertension (TA: 152/97 mmHg) and a distended abdomen with generalised tenderness and hypoactive bowel sounds. There was no fever, abdominal guarding, rebound or rigidity. Laboratory results were within normal limits. A plain radiograph of the abdomen revealed a “coffee bean” sign. We also observed an impressive picture of a typical “whirl” sign and a “bird's beak” sign on an emergent abdominal computed tomography (CT) scan. Also, his CT scan revealed marked distension and a twisted loop of sigmoid colon. Sigmoid volvulus (SV) was diagnosed rapidly with these characteristic radiological signs. Subsequently, with flexible sigmoidoscopy, the patient was successfully decompressed and detorsioned. SV is potentially life-threatening and requires emergency intervention. It is the third leading cause of colon obstruction in adults after cancer and diverticulitis. The primary emergency therapy for uncomplicated SV is endoscopic detorsion and decompression. Emergency physicians in particular should be aware of the typical radiographic CT signs, “coffee bean,” “whirl” and “bird's beak,” which are indicative of SV and which will allow them to easily diagnose this condition in cases of acute abdominal obstruction. If emergency physicians delay diagnosis, the patients might require emergency surgical intervention.

**Figure 1:**
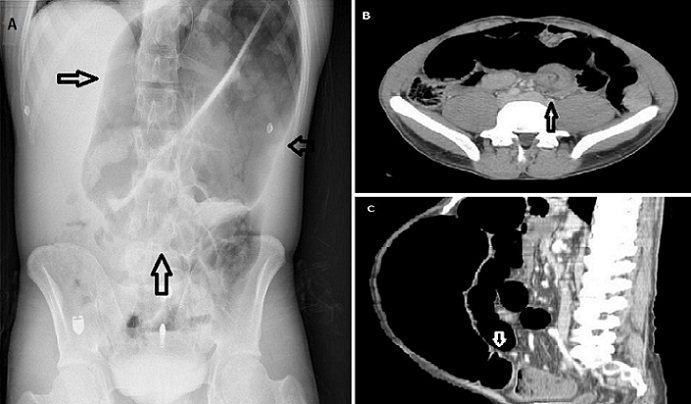
Coffee bean sign (A), whirl sign (B) and bird's beak sign (C) in the diagnosis of sigmoid volvulus

